# The Healing Effect of Sesame Oil, Camphor and Honey on Second Degree Burn Wounds in Rat

**Published:** 2018-01

**Authors:** Reza Vaghardoost, Seyed GholamReza Mousavi Majd, Hamid Tebyanian, Hamid Babavalian, Leila Malaei, Mitra Niazi, Ali Javdani

**Affiliations:** 1 Shahid Motahari Burn Hospital, Iran University of Medical Sciences, Tehran, Iran;; 2 Nanobiotechnology Research Center, Baqiyatallah University of Medical Sciences, Tehran, Iran;; 3 Applied Virology Research Center, Baqiyatallah University of Medical Sciences, Tehran, Iran

**Keywords:** Sesame oil, Camphor, Honey, Burn, Wound, Healing

## Abstract

**BACKGROUND:**

Many studies were carried out to improve sophisticated dressings to accelerate healing processes and reduce the microbial burden in burn wounds. This study evaluated the healing effect of herbal ointment containing extract of sesame oil, camphor and honey on second degree burn wounds in rats in comparison with daily dressing oil vaseline.

**METHODS:**

Forty rats were randomly assigned to two equal groups. A deep second degree burn was formed on the back of each rat with using a standard burning technique. The burns were dressed daily with herbal ointment containing extract of sesame oil, camphor and honey in group 1, dressing oil vaseline in group 2. The response to treatment was evaluated by digital photography during the treatment on 0, 7, 14, 21, 28 days. Histological scoring was undertaken for scar tissue samples on 0, 7, 14, 21, 28 days.

**RESULTS:**

Considerable epithelization in the herbal ointment group vs. the control group over the study period was noted. Neovascularization was significantly higher in herbal ointment treated rats as well. In terms of difference of wound surface area, maximal healing was noticed in herbal ointment extract of sesame oil, camphor and honey group and the minimal repair in the control group.

**CONCLUSION:**

The greatest rate of healing was in the herbal ointment group containing sesame oil, camphor and honey, so the herbal ointment as a suitable substitute for dressing and healing of burn wound injuries is recommended.

## INTRODUCTION

Burn is observed as one of the emergencies affecting both developed and developing countries leading to physical and psychological debilities with a growing trend in mortality and injury during pregnancy.^1^^-^^4^ Wound healing is extensively discussed in the medicinal literature. In current burn therapy, silver sulfadiazine was presented as the gold standard having antibacterial properties. Many studies were supported to develop more sophisticated dressings to accelerate healing procedure and reduce bacterial burden in wounds.^5^^-^^8^

Even medicinal plants were presented in wound healing of burned wounds, old-style forms of medicine, specifically herbal products, which have been used for centuries in the world and they are under scientific study for their characters in wound healing.^9^^-^^12^ There are numerous studies which they confirm the use of herbal plants for treatment of wounds. The mixed of sesame oil, as herbal medication, has been employed for debridement necrotic burns in our old-style medication.^13^

Honey is the oldest known treatments. Honey has been appreciated highly in the world and was declared in the Holy Quran since 1436 years ago. It has been employed for healing of respiratory diseases, urinary diseases, gastrointestinal diseases and skin diseases including ulcers, wounds, eczema, psoriasis and dandruff. Honey decreases inflammation, edema and exudation, promotes healing, diminishes the scar size and stimulates tissue regeneration.^14^^-^^16^ In this research, herbal ointment (composed of sesame oil, camphor and honey) were investigated on second degree burn wound in rat in comparison with vaseline. 

## MATERIALS AND METHODS 

In a randomized clinical trial, 40 Wistar-albino male rats (average weight: 300-350 g, average age: 3-4 months) were randomly divided into 2 equal groups. Group 1 received herbal ointment containing the extract of sesame oil, camphor and honey; group 2 was treated with dressing of vaseline. They were all maintained in a sheltered environment (temperature: 20-25˚C and humidity: 65-75%) under the supervision of a veterinarian. During the experiment, the rats were fed with usual rat chow and tap water and each group was kept in a separate cage. Studies on all groups were done at the same time. The rats were anesthetized by xylazine (10 mg/kg) and ketamine hydrochloride (50100 mg/kg) as described before.^9^^,^^14^^,^^17^^,^^18^ It should be noted that all surgical procedures and maintenance condition on laboratory animals had been done in accordance with the ethics committee. 

A standard second degree burn wound was formed with using a hot plate with the same size about 20% total body surface area and at identical temperature.^18^ Briefly, the skin on the dorsum was shaved with an electrical clipper. A deep second-degree burn wound was created with a hot plate (diameter: 4×2 cm) at an identical temperature (warmed for 5 min in hot water and placed for 10 sec on the skin with an equal pressure). The burn area was treated with the herbal ointment in group 1, and vaseline in group 2. Response to treatment was evaluated by digital photography on days 0, 7, 14, 21, and 28 under general anesthesia during the treatment.^9^^,^^14^^,^^17^^,^^18^

Histologic criteria were considered to determine efficacy of treatment including epithelization and neovascularization. In addition, speed of wound contraction was evaluated on days 0, 7, 14, 21, and 28. Every sample was taken under general anesthesia by a punch device which contained a part of burn wound and its surrounding skin.^6^^,^^8^^,^^16^^,^^18^ SPSS software (Version 2, Chicago, IL, USA) was used for statistical analysis. Non-parametric Friedman test was used to compare the groups. The level of statistical were considered significant difference (*p*<0.05). Measurement of treatment size between two groups were done by Image J software. 

## Results

**Fig. 1:**
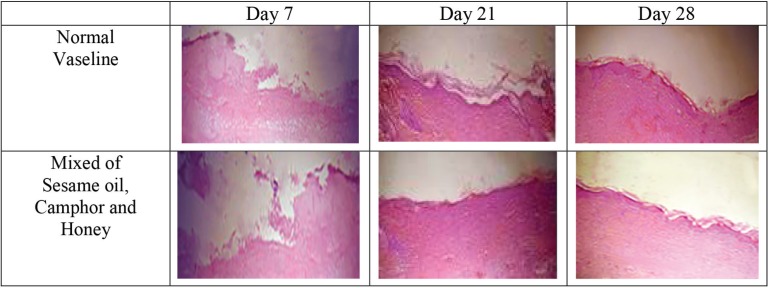
Histological Epithelization (×40).

**Fig. 2:**
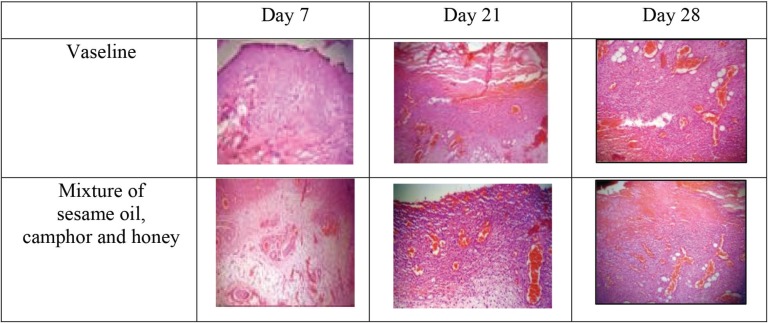
Histological neovascularization (×40).

**Fig. 3:**
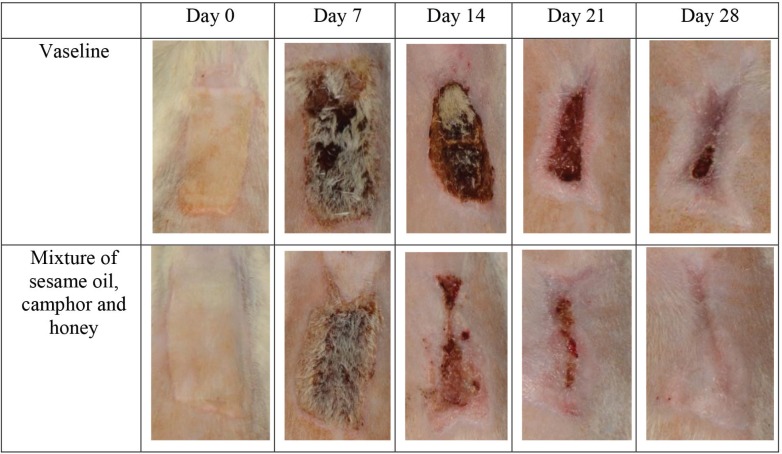
Treatment between two groups.

**Fig. 4:**
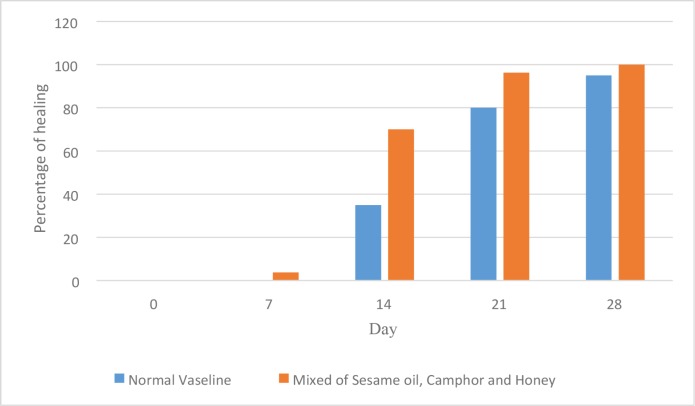
The percentage of wound healing in second degree burns.

There was a considerable epithelization in the vaseline group on days 7, 21, and 28, compared with the herbal ointment group (Figure 1). In addition, neovascularization was significantly more in the herbal ointment group than the vaseline group on days 7, 21, and 28 and the histological findings were demonstrated in Figure 2. There was not any significant difference between groups regarding the time of burn wound contraction on days 0, 7, 14, 21, and 28 and the results were exhibited in Figure 3 and Table 1. The percentage of wound healing was presented in Figure 4. 

**Table 1:** Measurement of treatment size between two groups.

**Variable**	**Day 0 **	**Day 7 **	**Day 14 **	**Day 21 **	**Day 28 **
Vaseline	8	8	5/2	1/6	0/4
Mixture of sesame oil, camphor and honey	8	7/7	2/4	0/3	0

Our results are based on three independent experiments. Nonparametric Friedman test, *p*<0.05.

## Discussion

Burns are the most common injuries, specifically among children. Most people can recover from burns without serious health consequences, depending on the cause and degree of injury. More serious burns need direct emergency medical care to avoid of complications and death. Three primary types of burns are called: first, second, and third degree. Each degree is based on the harshness of injury to the skin, with first degree is the most minor and third-degree is the most severe. Damage contains: first degree burns: red, non-blistered skin, second-degree burns: blisters and some thickening of the skin, third-degree burns: widespread thickness with a white, leathery appearance. Second degree burns spread in beneath of epidermis and into the dermis.^9^^,^^14^^,^^16^^-^^18^

Many researchers investigated on second degree burns wound using *Arnebia euchroma* ointment versus silver sulfadiazine to be efficient in wound healing process.^17^ Afshar *et al*. showed that Emu oil can have a positive effect on burns wound healing.^19^ Hmmati *et al*. showed the healing effect of n-hexan dichloromethane extract root *Onosma bulbotrichum* in second degree burns.^18^ Khorasani *et al*. showed that saffron (*Crocus sativus*) could help the accelerating wound healing in second degree burn injuries.^20^ Gupta *et al* showed that honey dressings made the wounds sterile in less time, and increased the second degree burn wound healing.^14^ Akhoondinasab et al, found that wound healing was more noticeable in Aloe vera group and also, they showed that the speed of wound healing was better in Aloe vera group than silver sulfadiazine group.^21^

In this research, histological epithelization and neovascularization assessment showed that epithelization and neovascularization in vaseline group were less and the time of burn wound contraction was shorter in the herbal ointment group during mentioned times. The mixture of sesame oil, camphor and honey was assessed for first time on healing of second degree burn wound injuries in rat with successful results. So it can be concluded that herbal ointment of sesame oil, camphor and honey had a significant effect on epithelization and neovascularization. Also, the time of burn wound contraction was significant in this group that can be recommend in healing of second degree burn wound injuries. 
